# Exposure–response analysis of endoxifen serum concentrations in early-breast cancer

**DOI:** 10.1007/s00280-020-04089-x

**Published:** 2020-05-29

**Authors:** Anabel Beatriz Sanchez-Spitman, Dirk-Jan A. R. Moes, Jesse J. Swen, Vincent O. Dezentjé, Diether Lambrechts, Patrick Neven, Hans Gelderblom, Henk-Jan Guchelaar

**Affiliations:** 1 Department of Clinical Pharmacy and Toxicology, Leiden University Medical Center, Albinusdreef 2, 2300 RC Leiden, The Netherlands; 2 Leiden Network for Personalised Therapeutics, Leiden University Medical Center, Leiden, The Netherlands; 3 Department of Medical Oncology, Netherlands Cancer Institute/Antoni van Leeuwenhoek, Amsterdam, The Netherlands; 4 Center for Cancer Biology, VIB, Leuven, Belgium; 5 Laboratory for Translational Genetics, Department of Human Genetics, KU Leuven, Leuven, Belgium; 6 Department of Medical Oncology, University Hospital Leuven, Leuven, Belgium; 7 Department of Medical Oncology, Leiden University Medical Center, Leiden, The Netherlands

**Keywords:** Endoxifen, TDM, Tamoxifen, Clinical outcome, Breast cancer

## Abstract

**Purpose:**

Tamoxifen is part of endocrine therapy in breast cancer treatment. Studies have indicated the use of endoxifen concentrations, tamoxifen active metabolite, to guide tamoxifen efficacy. Three endoxifen thresholds have been suggested (5.9 ng/ml, 5.2 ng/ml and 3.3 ng/ml) for therapeutic drug monitoring (TDM). Our aim was to validate these thresholds and to examine endoxifen exposure with clinical outcome in early-breast cancer patients using tamoxifen.

**Methods:**

Data from 667 patients from the CYPTAM study (NTR1509) were available. Patients were stratified (above or below), according to the endoxifen threshold values for tamoxifen efficacy and tested by Cox regression. Logistic regressions to estimate the probability of relapse and tamoxifen discontinuation were performed.

**Results:**

None of the thresholds showed a statistically significant difference in relapse-free survival: 5.2 ng/ml threshold: hazard ratio (HR): 2.545, 95% confidence interval (CI) 0.912–7.096, *p* value: 0.074; 3.3 ng/ml threshold: HR: 0.728; 95% CI 0.421–1.258, *p* value: 0.255. Logistic regression did not show a statistically significant association between the risk of relapse (odds ratio (OR): 0.971 (95% CI 0.923–1.021, *p* value: 0.248) and the risk for tamoxifen discontinuation (OR: 1.006 95% CI 0.961–1.053, *p* value: 0.798) with endoxifen concentrations.

**Conclusion:**

Our findings do not confirm the endoxifen threshold values for TDM nor does it allow definition of a novel threshold. These findings indicate a limited value of TDM to guide tamoxifen efficacy.

## Introduction

In the therapy of breast cancer, tamoxifen has been successfully prescribed for more than 40 years as adjuvant endocrine therapy in early-breast cancer patients [1]. In the current clinical guidelines, tamoxifen is recommended for premenopausal female patients as a 5-year monotherapy [2, 3], whereas for postmenopausal women a switch to an aromatase inhibitor is advised after two of 3 years of tamoxifen treatment [2, 3].

**Fig. 1:**
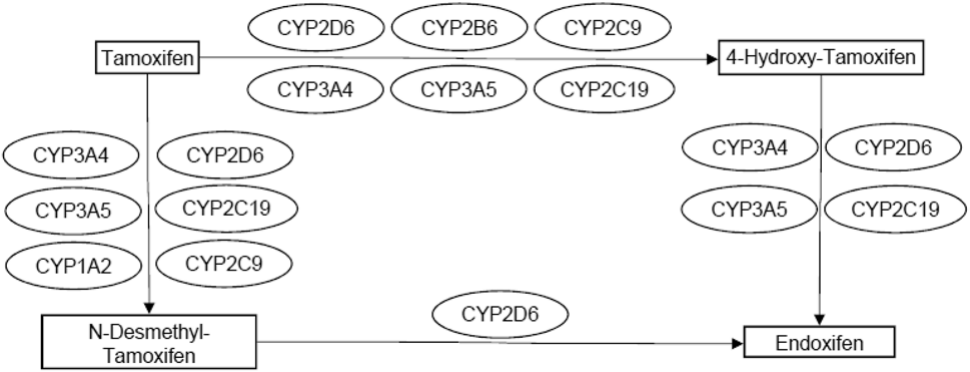
Tamoxifen metabolism

Tamoxifen is a selective estrogen receptor modulator that is characterised by a complex metabolism. Initially, tamoxifen is metabolised into its primary metabolites, *N*-desmethyl-tamoxifen (NDM-tamoxifen) and 4-hydroxy-tamoxifen, whilst a second conversion from NDM-tamoxifen and 4-hydroxy-tamoxifen leads to endoxifen (Fig. 1).

Among all tamoxifen metabolites, 4-hydroyx-tamoxifen and endoxifen are recognized as the active metabolites of tamoxifen. Both tamoxifen metabolites do have similar anti-estrogenic activity [4], being 30 to 100 times higher than the anti-estrogenic activity of the parent compound tamoxifen. However, endoxifen is considered the most important and the principal metabolite of tamoxifen metabolite, mostly because endoxifen is detected in 5 to 10 fold higher concentrations than 4-hydroxy-tamoxifen [5]. Interestingly, endoxifen’s mechanism of action might also differ from tamoxifen and its other metabolites, since it has been suggested to be concentration-dependent [6].

In the search for a more effective manner to predict tamoxifen efficacy in early-breast cancer patients, therapeutic drug monitoring (TDM) of endoxifen concentrations has been proposed [7]. To date, only a few studies have investigated the association between endoxifen concentrations and clinical outcomes in breast cancer patients receiving adjuvant tamoxifen. In the first research exploring this association, Madlensky et al. reported a threshold for endoxifen of 5.97 ng/ml [8]. According to these results, patients with an endoxifen concentration above this cutoff value, had at least a 26% decreased probability of breast cancer recurrence in comparison with patients with an endoxifen concentration below this threshold (adjusted hazard ratio (HR): 0.76, 95% confidence interval (CI) 0.55–1.00). For this retrospective analysis, the authors analysed a subset of 1370 women who were previously enrolled in the Women’s Healthy Eating and Living (WHEL) study and patients were stratified in five different endoxifen concentration groups. In this study, only blood samples after at least 4 months of tamoxifen treatment were retrieved.

Likewise, Saladores et al. reported a comparable threshold value for endoxifen concentration of 5.2 ng/ml in a study cohort of 306 premenopausal women [9]. In this study, patients were again divided into quartiles or four groups according to their endoxifen concentration and only when a comparison between the group with low endoxifen concentrations (< 5.2 ng/ml or < 14.15 nM) and the group with high endoxifen concentrations (> 12.9 ng/ml or > 35 nM) was made, a worsened clinical outcome, expressed as distant relapse-free survival, was observed (adjusted HR: 1.94; 95% CI 1.04–4.14).

In another study by Helland and colleagues, a much lower endoxifen threshold concentration of 3.3 ng/ml (or 9 nM) was related to poorer survival outcome [10] (adjusted HR: 3.70; 95% CI 1.03–13.25; *p* value: 0.029). In this study, 99 pre- and postmenopausal patients were investigated, with a median follow-up of 13.9 years. An important advantage of this study compared to other studies is the use of 4-hydroxy-tamoxifen concentrations for which a threshold for efficacy was reported. According to the authors, patients with a concentration of 4-hydroxy-tamoxifen below 3.26 nM, had worsened clinical outcomes when compared with those patients with higher 4-hydroxy-tamoxifen concentrations (Adjusted HR: 3.56; 95% CI 1.14–11.07; *p* value: 0.020).

Although all these studies focused on finding the lowest concentration levels of endoxifen associated with clinical outcome, Love and colleagues suggested an upper limit of 70 ng/ml for endoxifen concentrations above which patients might have a higher chance of cancer relapse [11]. Although these findings were obtained in a nested case–control cohort of only 48 patients, authors did not report a minimal endoxifen concentration for tamoxifen efficacy. In the same line, Groenland et al. did not find statistically significant differences of clinically important toxicities among patients with endoxifen concentration levels above 25 ng/ml compared to patients with lower endoxifen concentrations [12]. In contrast to Groenland, another study by Helland and colleagues [13] suggest that higher tamoxifen metabolite concentrations, may be associated with adverse effects, such as vaginal dryness. Of note, endoxifen concentration was not related to any of the analysed adverse effects.

All of these studies might also have limitations, such as the fact that their outcomes and conclusions were based on the retrospective cohorts of patients. An important difference across these studies are the number of patients and the different study populations. For instance, Saladores analysed only premenopausal women [9], whilst Helland [10] and Madlensky [8] studied both pre- and postmenopausal patients.

In contrast to these studies, a recent prospective study by Neven et al. in which 297 breast cancer patients receiving tamoxifen in the metastatic and neoadjuvant setting failed to identify a relationship between improved survival outcome and endoxifen concentrations [14]. In the same line, another recent research also in the metastatic scenario by Takano and colleagues [15] did not detect any association between endoxifen concentration levels and tamoxifen efficacy. In this study, authors enrolled 186 Japanese women between December 2012 and March 2016 diagnosed with stage IV breast cancer who received tamoxifen as first-line of treatment. In this study, authors concluded that no differences in the survival outcome, defined as progression-free survival, were observed (HR: 0.75, 95% CI 0.50–1.14).

Another recently published study performed in the adjuvant setting, followed 667 women diagnosed with early-breast cancer and treated with tamoxifen as adjuvant endocrine therapy were also evaluated. In this case, the putative association between *CYP2D6* genotypes and endoxifen concentrations with relapse-free survival was also investigated, but no differences in survival outcomes were obtained. Therefore, these outcomes were in line with to those of Neven and colleagues [14] and Takano et al. [15].

Owing to the differences across studies, the use of TDM of endoxifen for guiding individual tamoxifen treatment in the clinical practice is still not generally implemented [7] and disagreements in the interpretations regarding the conclusions of these studies are present [16–19].

Therefore, we aimed to examine the exposure–response relationship of endoxifen in a large prospective cohort of women with early-breast cancer using tamoxifen.

## Materials and methods

### Study population and design

To investigate the association of endoxifen concentrations with clinical outcomes, serum samples and clinical data, such as follow-up and clinical characteristics, from the CYPTAM cohort (NTR1509) of early-breast cancer patients treated with adjuvant tamoxifen were analysed. This study population of 667 patients was recruited between February 2008 and December 2010 in the Netherlands and Belgium. Shortly, the co-primary objectives of this observational study were to evaluate the association of endoxifen serum concentrations and CYP2D6 predicted phenotypes with breast cancer relapse. According to the inclusion criteria, only female early-breast cancer patients receiving 20 mg QD adjuvant tamoxifen could be included. In addition, patients who were already using tamoxifen but for less than 12 months from the start of the treatment were eligible. In all cases, a serum sample from each included patient for measuring the concentrations of tamoxifen, NDM-tamoxifen, 4-hydroxy-tamoxifen and endoxifen were retrieved at least 2 months after the start of the treatment with tamoxifen to assure steady-state concentrations. Of note, 24 patients of this study population participated in another separated study in which a temporary (2 months) increase in tamoxifen doses were used. However, we did not take the temporary increase of the dose into account as we considered it as neglectable as compared to the median duration of standard dose of daily 20 mg of tamoxifen [20].

All patients gave written informed consent. The Institutional Review Board of the Leiden University Medical Center approved the study protocol. A more detailed description of CYPTAM has been published previously [16, 21, 22].

### Study objectives

The primary objective of the current analysis was to examine the impact of the all proposed threshold for endoxifen serum concentrations from the literature (5.9 ng/ml [8], 5.2 ng/ml [9] and 3.3 ng/ml [10]) and the median endoxifen serum concentration (10.3 ng/ml) in a prospectively designed study with a large cohort of female breast cancer patients using tamoxifen and who previously were enrolled in the CYPTAM study [16]. This median concentration value for endoxifen was selected in order to uniformly assess the exposure to anti-estrogenic activity of endoxifen in this study population. In addition, patients were categorized in quartiles, according to their endoxifen concentration levels. Of note, outcomes of the survival analysis for the endoxifen threshold of 5.9 ng/ml and endoxifen as a continuous variable (accounting from the start of tamoxifen treatment) were already reported as an exploratory analysis in the CYPTAM study [16]. In the current manuscript, they are presented again for a comparison to all the thresholds for endoxifen concentrations available in the literature.

For the purpose of this study, relapse-free survival was chosen as the primary endpoint. RFSt was described as the time from initiation of tamoxifen treatment until loco-regional or distant relapse or secondary breast cancer. If a patient switched to an aromatase inhibitor after 2 or 3 years of tamoxifen treatment, censoring at the time of tamoxifen discontinuation occurred, as previously also analysed in the CYPTAM study [16].

The secondary objectives were to investigate the effect of endoxifen concentrations and its relationship with the probability of breast cancer relapse and of tamoxifen discontinuation in the same study population.

### Measurement of tamoxifen and its metabolites concentrations

Tamoxifen, NDM-tamoxifen, 4-hydroxy-tamoxifen and endoxifen through concentrations were measured in serum at steady state (> 2 months after start of tamoxifen).

Concentrations of tamoxifen and its three metabolites were quantified by a high-performance liquid chromatography–tandem mass spectrometry (HPLC–MS/MS). This method was developed and validated according to the EMA bioanalytical method validation guideline by the Clinical Pharmacy and Toxicology Department of Leiden University Medical Center in line with a previously described bioanalytical method [23].

### Statistical analysis

For the primary objective, all patients were divided in two groups according to their endoxifen steady-state concentrations (Endoxifen threshold 5.9 ng/ml: ≤ 5.9 ng/ml vs> 5.9 ng/ml; Endoxifen threshold 5.2 ng/ml: ≤ 5.2 ng/ml vs> 5.2 ng/ml; Endoxifen threshold 3.3 ng/ml: ≤ 3.3 ng/ml vs> 3.3 ng/ml; Median endoxifen concentration 10.3 ng/ml: ≤ 10.3 ng/ml vs> 10.3 ng/ml). To evaluate differences of the patient’s demographics across groups, χ2 tests or t statistics or Mann–Whitney tests was performed used, depending on the type of data.

For the analysis of the primary objective, Cox regression was performed to analyse whether RFSt differed through all the four groups (Hazard Ratios; HR). For this analysis, uni- and multivariable analysis applied. In the case of univariable analysis, when a *p* value < 0.1 was obtained, this covariate was adopted in the multivariable analysis. Yet, the following covariates were fitted in the multivariable analysis due to their clinical relevance: tumour and nodal stage, histological classification and grade and Her2 receptor status.

For the secondary objective, a logistic regression analysis was performed. Because our aim was to depict how the probability of breast cancer recurrence varies according to endoxifen concentrations, the use of a logistic model was required. In the same manner, another logistic regression was performed to evaluate the chance of discontinuation of tamoxifen treatment related to endoxifen concentrations. In this case, treatment discontinuation with tamoxifen due to side effects was used as a proxy to estimate the effect of side effects. For both analyses, odds ratios (OR) were calculated in order to determine the effect size. All statistical analyses were performed with IBM SPSS for Windows, Version 23.0 and R studio Version 1.0.456 and package R (v3.4.4). Also, statistical significance was accepted for *p* values below 0.05.

## Results

### Study population

In total, 667 breast cancer patients who were receiving adjuvant tamoxifen were included in the CYPTAM study. A more comprehensive overview of the demographic characteristics is presented elsewhere [16, 21, 22, 24].

For this study, patients were categorized in the different groups depending on their endoxifen serum concentration according to the different proposed endoxifen thresholds (5.9 ng/ml, 5.2 ng/ml, 3.3 ng/ml and median (10.3 ng/ml). Of note, patients with endoxifen concentrations below the 5.9 ng/ml, 5.2 ng/ml, 3.3 ng/ml and 10.3 ng/ml threshold were 139 (21%), 112 (16.9%), 49 (7.4%) and 332 (50.2%) patients, respectively. At baseline, no differences in clinical characteristics were observed (*p* > 0.05) (Table 1), with the exception of the progesterone receptor status (positive or negative) and axillar surgery (sentinel node procedure only or axillary lymph node dissection) in the group of endoxifen threshold of 3.3 ng/ml. The median follow-up was 6.8 years (range 0.33–9.34 years) and the total event rate during tamoxifen therapy was 8.5%. As previously reported [16], approximately 66% of the enrolled patients started tamoxifen as endocrine therapy and switched to an aromatase inhibitor after two or three years of endocrine therapy.

**Table 1:** Demographic characteristics of the CYPTAM study population categorized by the different proposed endoxifen threshold (5.9 ng/ml, 5.2 ng/ml, 3.3 ng/ml and Median (10.3 ng/ml) according to endoxifen serum concentrations

	Endoxifen threshold of 5.9 ng/ml			Endoxifen threshold of 5.2 ng/ml			Endoxifen threshold of 3.3 ng/ml			Endoxifen threshold of 10.3 ng/ml (Median)		
	< 5.9 ng/ml	> 5.9 ng/ml	*p* value	< 5.2 ng/ml	> 5.2 ng/ml	*p*-value	< 3.3 ng/ml	> 3.3 ng/ml	*p*-value	< 10.3 ng/ml	> 10.3 ng/ml	*p*-value
	*N* (%)	*N* (%)		*N* (%)	*N* (%)		*N* (%)	*N* (%)		*N* (%)	*N* (%)	
Age at tamoxifen initiation												
Mean in years (SD)	55.4 (10.3)	56.7 (11.3)	0.219	55.4 (10.6)	56.6 (11.2)	0.302	57.3 (9.9)	56.3 (11.2)	0.543	55.4 (10.6)	57.5 (11.4)	0.015
Tumour stage												
T1	77 (55.4%)	277 (53.0%)	0.052	61 (54.5%)	293 (53.3%)	0.132	28 (57.1%)	326 (53.2%)	0.712	180 (54.2%)	174 (52.7%)	0.757
T2	61 (43.9%)	211 (40.3%)		50 (44.6%)	222 (40.4%)		20 (40.8%)	252 (41.1%)		135 (40.7%)	137 (41.5%)	
T3/T4	1 (0.7%)	27 (5.2%)		1 (0.9%)	27 (4.9%)		1 (2.0%)	27 (4.4%)		12 (3.6%)	16 (4.8%)	
Not specified	0 (0.0%)	8 (1.5%)		0 (0.0%)	8 (1.5%)		0 (0.0%)	8 (91.3%)		5 (1.5%)	3 (0.9%)	
Nodal stage												
N0	68 (48.9%)	247 (47.2%)	0.726	54 (48.2%)	261 (47.5%)	0.872	27 (55.1%)	288 (47.0%)	0.828	146 (44.0%)	169 (51.2%)	0.300
N1	52 (37.4%)	213 (40.7%)		42 (37.5%)	223 (40.5%)		17 (34.7%)	248 (40.5%)		139 (41.9%)	126 (38.2%)	
N2	15 (10.8%)	42 (8.0%)		12 (10.7%)	45 (8.2%)		4 (8.2%)	53 (8.6%)		31 (9.3%)	26 (7.9%)	
N3	4 (2.9%)	19 (3.6%)		4 (3.6%)	19 (3.5%)		1 (2.0%)	22 (3.6%)		15 (4.5%)	8 (2.4%)	
Not specified	0 (0.0%)	2 (0.4%)		0 (0.0%)	2 (0.4%)		0 (0.0%)	2 (0.3%)		1 (0.3%)	1 (0.3%)	
Histological classification												
Ductal adenocarcinoma	105 (75.5%)	399 (76.3%)	0.887	87 (77.7%)	417 (75.8%)	0.876	40 (81.6%)	464 (75.7%)	0.775	258 (77.7%)	246 (74.5%)	0.820
Lobular adenocarcinoma	21 (15.1%)	73 (14.0%)		16 (14.3%)	78 (14.2%)		6 (12.2%)	88 (14.4%)		44 (13.3%)	50 (15.2%)	
Others	13 (9.4%)	49 (9.4%)		9 (8.0%)	53 (9.6%)		3 (6.1%)	59 (9.6%)		29 (8.7%)	33 (10.0%)	
Not specified	0 (0.0%)	2 (0.4%)		0 (0.0%)	2 (0.4%)		0 (0.0%)	2 (0.3%)		1 (0.3%)	1 (0.3%)	
Histological grade												
G1	17 (12.2%)	76 (14.5%)	0.893	13 (11.6%)	80 (14.5%)	0.876	7 (14.3%)	86 (14.0%)	0.922	48 (14.5%)	45 (13.6%)	0.808
G2	80 (57.6%)	297 (56.8%)		65 (58.0%)	312 (56.7%)		28 (57.1%)	349 (56.9%)		185 (55.7%)	192 (58.2%)	
G3	41 (29.5%)	145 (27.7%)		33 (29.5%)	153 (27.8%)		14 (28.6%)	172 (28.1) %		95 (28.6%)	91 (27.6%)	
Not specified	1 (0.7%)	5 (1.0%)		1 (0.9%)	5 (0.9%)		0 (0.0%)	6 (1.0%)		4 (1.2%)	2 (0.6%)	
Progesterone receptor status												
Positive	112 (80.6%)	415 (79.3%)	0.297	92 (82.1%)	435 (79.1%)	0.361	46 (93.9%)	481 (78.5%)	0.035	266 (80.1%)	261 (79.1%)	0.060
Negative	27 (19.4%)	99 (18.9%)		20 (17.9%)	106 (19.3%)		3 (6.1%)	123 (20.1%)		65 (19.6%)	61 (18.5%)	
Not specified	0 (0.0%)	9 (1.7%)		0 (0.0%)	9 (1.6%)		0 (0.0%)	9 (1.5%)		1 (0.3%)	8 (2.4%)	
HER2 Neu receptor status												
0	85 (61.2%)	320 (61.2%)	0.961	68 (60.7%)	337 (61.3%)	0.821	29 (59.2%)	376 (61.3%)	0.755	204 (61.4%)	201 (60.9%)	0.716
1+	35 (25.2%)	131 (25.0%)		31 (27.7%)	135 (24.5%)		15 (30.6%)	151 (24.6%)		82 (24.7%)	84 (25.5%)	
2+	8 (5.8%)	27 (5.2%)		4 (3.6%)	31 (5.6%)		1 (2.0%)	34 (5.5%)		21 (6.3%)	14 (4.2%)	
3+	11 (7.9%)	43 (8.2%)		9 (8.0%)	45 (8.2%)		4 (8.2%)	50 (8.2%)		24 (7.2%)	30 (9.1%)	
Not specified	0 (0.0%)	2 (0.4%)		0 (0.0%)	2 (0.4%)		0 (0.0%)	2 (0.3%)		1 (0.3%)	1 (0.3%)	
FISH												
Positive (amplification)	14 (10.1%)	45 (8.6%)	0.667	10 (8.9%)	49 (8.9%)	0.815	4 (8.2%)	55 (9.0%)	0.905	28 (8.4%)	31 (9.4%)	0.910
Negative	125 (89.9%)	476 (91.0%)		102 (91.1%)	499 (90.7%)		45 (91.8%)	556 (90.7%)		303 (91.3%)	298 (90.3%)	
Not specified	0 0.0%)	2 (0.4%)		0 (0.0%)	2 (0.4%)		0 (0.0%)	2 (0.3%)		1 (0.3%)	1 (0.3%)	
Surgery												
Mastectomy	65 (46.8%)	243 (46.5%)	0.978	54 (48.2%)	254 (46.2%)	0.832	23 (46.9%)	285 (46.5%)	0.851	165 (49.7%)	143 (43.3%)	0.139
Breast conserving	73 (52.5%)	277 (53.0%)		57 (50.9%)	293 (53.3%)		26 (53.1%)	324 (52.9%)		164 (49.4%)	186 (56.4%)	
Not specified	1 (0.7%)	3 (0.6%)		1 (0.9%)	3 (0.5%)		0 (0.0%)	4 (0.7%)		3 (0.9%)	1 (0.3%)	
Surgery-axilla												
Sentinal node procedure only	67 (48.2%)	263 (50.3%)	0.897	54 (48.2%)	276 (50.2%)	0.858	26 (53.1%)	304 (49.6%)	0.778	147 (44.3%)	183 (55.5%)	0.012
Axillary lymph node dissection	71 (51.1%)	257 (49.1%)		57 (50.9%)	271 (49.3%)		23 (46.9%)	305 (49.8%)		182 (54.8%)	146 (44.2%)	
Not specified	1 (0.7%)	3 (0.6%)		1 (0.9%)	3 (0.5%)		0 (0.0%)	4 (0.7%)		3 (0.9%)	1 (0.3%)	
Adjuvant chemotherapy												
Yes	90 (64.7%)	315 (60.2%)	0.500	74 (66.1%)	331 (60.2%)	0.433	26 (53.1%)	379 (61.8%)	0.427	211 (63.6%)	194 (58.8%)	0.452
No	49 (35.3%)	206 (39.4%)		38 (33.9%)	217 (39.5%)		23 (46.9%)	232 (37.8%)		120 (36.1%)	135 (40.9%)	
Not specified	0 (0.0%)	2 (0.4%)		0 (0.0%)	2 (0.4%)		0 (0.0%)	2 (0.3%)		1 (0.3%)	1 (0.3%)	
Adjuvant radiotherapy												
Yes	104 (74.8%)	356 (68.1%)	0.560	80 (71.4%)	380 (69.1%)	0.741	35 (71.4%)	425 (69.3%)	0.889	234 (70.5%)	226 (68.5%)	0.855
No	35 (25.2%)	165 (31.5%)		32 (28.6%)	168 (30.5%)		14 (28.6%)	186 (30.3%)		97 (29.2%)	103 (31.2%)	
Not specified	0 (0.0%)	2 (0.4%)		0 (0.0%)	2 (0.4%)		0 (0.0%)	2 (0.3%)		1 (0.3%)	1 (0.3%)	
Trastuzumab treatment												
Yes	12 (8.6%)	46 (8.8%)	0.510	10 (8.9%)	48 (8.7%)	0.598	4 (8.2%)	54 (8.8%)	0.806	26 (7.8%)	32(9.7%)	0.270

*SD* standard deviation

### Analysis of endoxifen serum concentrations and the relationship with clinical outcome (RFSt)

The association between endoxifen serum concentrations, examined as continuous variable, with clinical outcome, evaluated as RFSt since the exposure to endoxifen (did not yield any significant differences in both uni- (hazard ratio (HR): 0.988, 95% confidence interval (CI) 0.944–1.035, *p* value: 0.613) and multivariable analysis (adjusted HR: 0.985, 95% CI 0.938–1.034, *p* value: 0.541) (Table 2). Interestingly, these results minimally vary from the previously described outcomes, in which the reported exposure was assessed from the time of enrolment [16]. At the same time, dividing all patients according to their endoxifen concentration in quartiles, did not change these outcomes (Table 2). For the primary objective of this study, the following proposed analyses were to estimate the usefulness of the different endoxifen threshold concentrations from the literature (5.9 ng/ml, 5.2 ng/ml and 3.3 ng/ml) and the endoxifen median concentration (10.3 ng/ml) of the CYPTAM study. A total of 4 groups were made, according to the endoxifen serum concentrations: below and above of 5.9 ng/ml, below and above of 5.2 ng/ml, below and above of 3.3 ng/ml and below and above of 10.3 ng/ml.

**Table 2:** Cox proportional hazards ratio model of RFSt

Endoxifen analysis	*N* (%)	Univariable analysis			Multivariable analysis*		
		HR	95% CI	*P*-value	HR	95% CI	*P*-value
Endoxifen** (ng/ml) (continuous variable)	662 (100)	0.988	0.944–1.035	0.613	0.985	0.938–1.034	0.541
Endoxifen concentration by quartile							
Q1: < 6.6 ng/ml	165 (24.9%)	1.000	Reference	0.319	1.000	Reference	0.181
Q2: 6.6–10.3 ng/ml	167 (25.2%)	1.748	0.817–3.739	0.150	1.879	0.864–4.090	0.112
Q3: 10.3–14.1 ng/ml	165 (24.9%)	1.311	0.582–2.954	0.513	1.272	0.553–2.927	0.571
Q4: > 14.1 ng/ml	165 (24.9%)	0.951	0.403–2.241	0.908	0.867	0.363–2.069	0.747
Endoxifen threshold**							
< 5.9 ng/ml	139 (21%)	1.000	Reference		1.000	Reference	
> 5.9 ng/ml	523 (79%)	1.382	0.652–2.928	0.399	1.426	0.666–3.053	0.361
Endoxifen threshold							
< 5.2 ng/ml	112 (16.9%)	1.000	Reference		1.000	Reference	
> 5.2 ng/ml	550 (83.1%)	2.391	0.863–6.621	0.094	2.545	0.912–7.096	0.074
Endoxifen threshold							
< 3.3 ng/ml	49 (7.4%)	1.000	Reference		1.000	Reference	
> 3.3 ng/ml	613 (92.6%)	3.508	0.485–25.378	0.214	2.992	0.410–21.822	0.280
Endoxifen threshold							
< 10.3 ng/ml	332 (50.2%)	1.000	Reference		1.000	Reference	
> 10.3 ng/ml	330 (49.8%)	0.803	0.472–1.365	0.418	0.728	0.421–1.258	0.255

*Adjusted for: Her2Neu status, histologic grade and classification, tumour size and nodal stage

**Outcomes presented the original CYPTAM study, and are reported here for completeness [1]. *Q1* quartile 1, *Q2* quartile 2, *Q3* quartile 3, *Q4* quartile 4, *RFSt* relapse-free survival during tamoxifen treatment

In the first analysis (below and above of 5.9 ng/ml), no statistically significant differences were found in either the uni-variable (HR: 1.382, 95% CI 0.652–2.928, *p* value: 0.399) or multivariable analysis (adjusted HR: 1.426, 95% CI 0.666–3.053, *p* value: 0.361). Similarly, using the endoxifen thresholds of 5.2 ng/ml and 3.3 ng/ml also did not relate to improve these outcomes, since the multivariate Cox analysis of HR 2.545 (95% CI 0.912–7.096, *p* value: 0.074) and HR 2.992 (95% CI 0.410–21.822.216, *p* value: 0.280) also failed to find an association, respectively. Also, dividing patients according to the endoxifen concentration and using the median endoxifen concentrations (10.3 ng/ml) of the CYPTAM study as cutoff point, were not associated with RFSt (univariate analysis: HR: 0.803, 95% CI 0.472–1.365, *p* value:0.418; adjusted HR: 0.728, 95% CI 0.421–1.258, *p* value: 0.255) (Table 2).

**Fig. 2:**
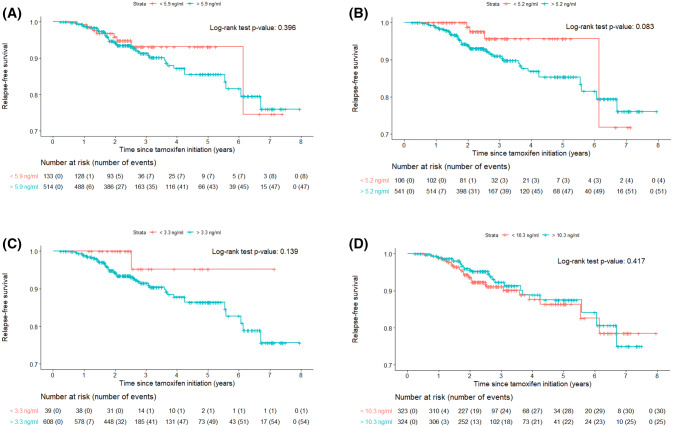
Kaplan–Meier representations of the proposed threshold for endoxifen concentrations: **a** 5.9 ng/ml; **b** 5.2 ng/ml; **c** 3.3 ng/ml; **d** 10.3 ng/ml

In accordance with the Cox regression analysis, none of the Kaplan–Meier analyses (log-rank) using any of the four endoxifen concentrations differed significantly (5.9 ng/ml: *p* value: 0.396; 5.2 ng/ml: *p* value: 0.083; 3.3 ng/ml; *p* value: 0.139; 10.3 ng/ml: *p* value: 0.417) (Fig. 2).

### Clinical outcome and endoxifen serum concentrations: logistic regression analysis

**Fig. 3:**
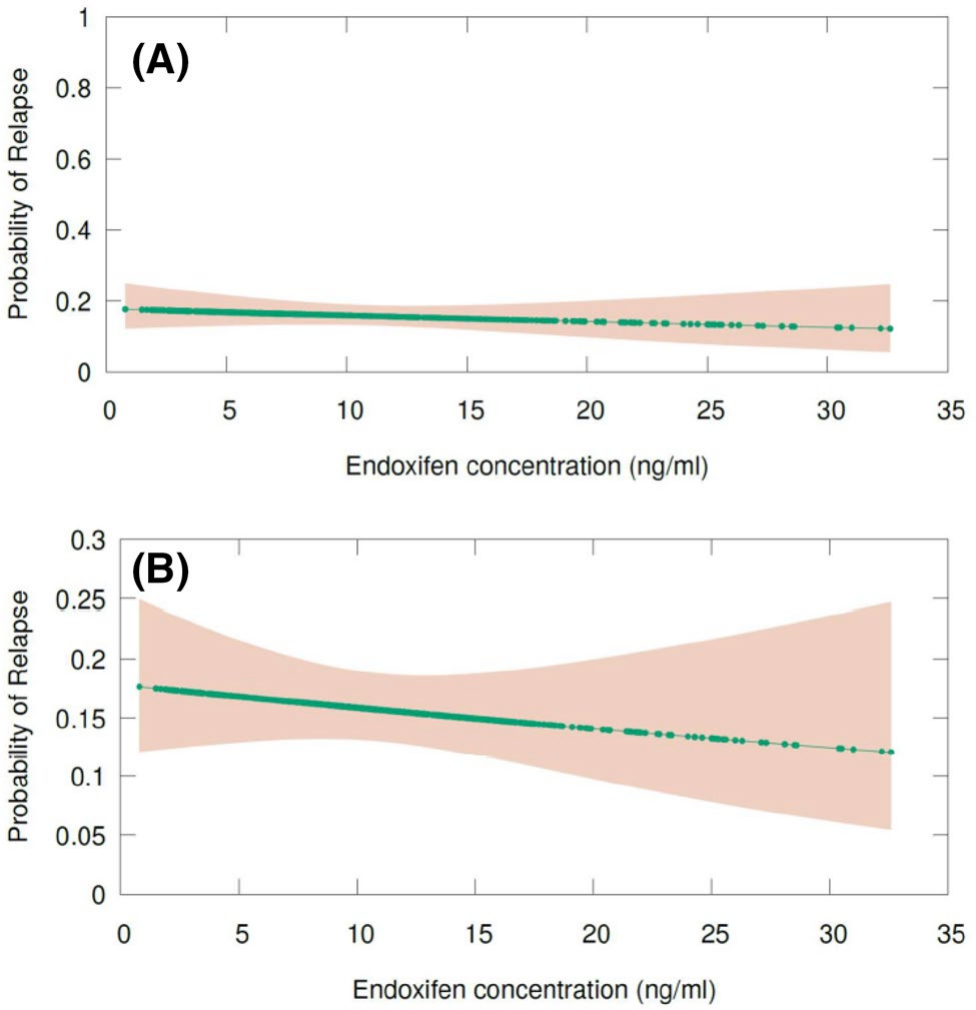
Logistic regression of the probability of relapse and endoxifen concentrations. **a** Probability of relapse and endoxifen concentrations (scale of probability 0–1). In this Figure, contrasting the probability of relapse against endoxifen concentrations leads to an almost flat line. **b** Probability of relapse and endoxifen concentrations (scale of probability 0–0.3). In this Figure, contrasting the probability of relapse against endoxifen concentrations shows a decreasing line

To evaluate the concentration effect of endoxifen and RFSt relationship, we adopted a different approach. For this analysis, the probability of relapse (relapse or no relapse) for each patient in the study population was calculated by performing a logistic regression analysis. Thereafter, these calculated probabilities were contrasted against the individual value of endoxifen concentrations of each patient. Interestingly, a decreasing line (illustrated with its 95% confidence interval) is observed: although the probabilities of relapse are slightly higher in the patients with a low endoxifen concentrations, a slightly lower chance of relapse is observed across the patients with higher endoxifen concentrations. In terms of effect size, the calculated OR was 0.971 (95% CI 0.923–1.021, *p* value: 0.248). Although this OR is not statistically significant, a visual representation may suggest a minor concentration–effect relationship for endoxifen levels and probability of relapse. This decrease of the probability of breast cancer relapse by higher endoxifen concentrations might roughly account for 5% in the probability of breast cancer recurrence. This logistic regression line is presented as Fig. 3.

### Tamoxifen discontinuation and endoxifen serum concentrations: logistic regression analysis

**Fig. 4:**
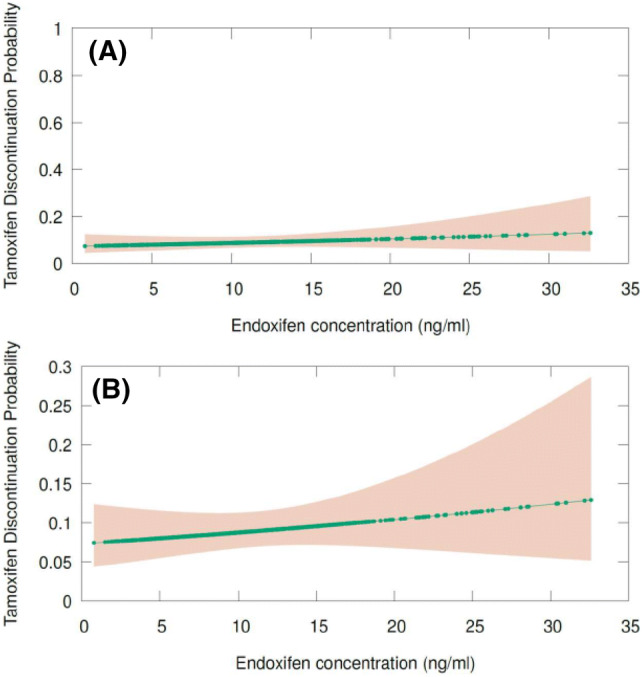
Logistic regression of the probability of tamoxifen discontinuation and endoxifen concentrations. **a** Probability of tamoxifen discontinuation and endoxifen concentrations (scale of probability 0–1). In this figure, contrasting the probability of tamoxifen discontinuation against endoxifen concentrations leads to roughly flat line. **b** Probability of tamoxifen discontinuation and endoxifen concentrations (scale of probability 0–0.3)

Next, we used an analogous approach to assess the concentration effect of endoxifen concentrations and the probability of tamoxifen discontinuation due to side effects by performing a second logistic regression analysis. To this end, we computed the probability of tamoxifen treatment discontinuation (stopping treatment with tamoxifen or not) for each individual in the enrolled CYPTAM cohort. In the same way, all these probabilities of tamoxifen discontinuation were compared and delineated against the endoxifen concentrations of every patient. In this analysis, the obtained OR was 1.006 (95% CI 0.961–1.053, *p* value: 0.798). In contrast to the previous analysis, an increasing line is depicted: whilst the probabilities of tamoxifen treatment discontinuation is minimally increased in patients with the highest endoxifen concentrations, a minor lower tamoxifen discontinuation probability is seen among patients with the lowest endoxifen concentrations. A presentation of this illustration is shown in Fig. 4.

## Discussion

In this large cohort of early-breast cancer patients receiving tamoxifen, logistic regression analyses suggest a minor exposure–response relation with a slightly decreased risk of relapse and a small increased risk for tamoxifen discontinuation at higher endoxifen concentrations. These observations indicates the existence of a concentration–effect relationship for endoxifen concentrations and the probability of breast cancer relapse (RFSt) however the clinical relevance seems limited. At the same time, using the proposed endoxifen concentration thresholds from the literature (5.9 ng/ml, 5.2 ng/ml and 3.3 ng/ml) and the median endoxifen concentration of 10.3 ng/ml were not associated with clinical outcome defined as RFSt.

Endocrine therapy with tamoxifen has been the standard-of-care for more than 40 years for women in the adjuvant and metastatic setting [25]. In the search of a biomarker to predict tamoxifen efficacy, alternatives such as endoxifen or 4-hydroxy-tamoxifen concentrations have been proposed [7, 10]. In the case of endoxifen concentrations, the 5.97 ng/ml threshold of Madlensky and colleagues is considered the most important cutoff point, whilst it is also the most widely used one [7]. However, we believe the application of this concentration in the current practice should be carefully evaluated. In their manuscript, Madlensky et al. did not report the used dose of tamoxifen neither endocrine therapy duration. At the same time, all described survival outcomes, e.g. Cox regression analysis, were analysed as disease-free survival, which was defined as the time of diagnosis till the time of second breast cancer. Since no additional information regarding tamoxifen exposure was included in their analysis (e.g. dose or therapy duration), this reported endoxifen concentration of 5.97 ng/ml may not correctly illustrate the impact of the exposure to endoxifen concentration. Another potential remark might be the minor difference in the percentages of recurrences observed across the studied groups (quintiles): while the percentage of recurrence of the lowest group (quintile) was 16%, this rate in the higher groups (quintiles) could be seen as comparable (e.g. recurrence rate in third quintile was 14.7%).

Although currently the majority of clinical guidelines recommends at least 5 years of endocrine therapy (either as tamoxifen or as aromatase inhibitor, or any of these combined) [2, 3], different strategies, e.g. 2 years vs 5 years of tamoxifen [26], were still suggested to be beneficial during the WHEL study. Consequently, quantifying this putative cutoff point for endoxifen in current antiestrogenic strategies may be extremely difficult to measure. In any case, we did not find any difference in our study when comparing both groups (above vs below 5.9 ng/ml) (adjusted HR: 1.426, 95% CI 0.666–3.053, *p* value: 0.361). Although the main advantage of Madlensky’s study might rely on the high number of included patients (1370 individuals), we also failed to find any association despite of analysing from the exposure to tamoxifen therapy. A possible reason for these outcomes might be due to the use of the term “threshold effect”.

Generally, a threshold effect is supposed to be an inflexion mark or level at which a significant variation takes place [27]. As observed in our proposed figure for endoxifen concentration and probability of relapse, around 5.9 ng/ml [8] or 5.2 ng/ml [9], or the even lower level of 3.3 ng/ml [10], no changes in the curve of our analysis could be found (Fig. 3). In contrast, we observed a decreasing curve in which higher endoxifen concentrations are related to lower probability of breast cancer relapse, suggesting a concentration effect relationship for endoxifen (Fig. 3). Interestingly, this lower chance of probability (around 5%) is in line with the main advantage of the use of adjuvant endocrine therapy with tamoxifen in terms of survival outcome [28]. At the same time, we also observed a growing line when contrasting the effect of endoxifen concentrations with the probability of treatment discontinuation with tamoxifen (Fig. 4).

In our opinion, whilst the hypothesis of lower probability of relapse by higher endoxifen concentrations might be plausible, we also showed that the chance of tamoxifen treatment discontinuation might be higher at higher endoxifen concentrations (Figs. 3 and 4). Consequently, using only endoxifen concentrations as a proxy for tamoxifen efficacy, should be considered cautiously. Owing to the higher endoxifen concentrations (e.g. due to a higher dose of tamoxifen), patients also could tend to have a higher chance of treatment discontinuation due to side effects and therefore, lower adherence, which could potentially lead to treatment failure.

In order to improve the prediction of tamoxifen efficacy, we consider that the anti-estrogenic activity of tamoxifen might not only rely on endoxifen concentrations, but many other variables, e.g. other tamoxifen metabolites and their concentrations, might also be responsible for this difference in relapse. For instance, 4-hydroxy-tamoxifen has an anti-estrogenic activity similar to endoxifen [4], but endoxifen has always been contemplated as the most active metabolite of tamoxifen, since it is found in higher concentrations than 4-hydroxy-tamoxifen [5]. Another example of a difference approach based on the concentrations of other tamoxifen metabolites instead of only endoxifen concentrations was described by De Vries-Schultink and colleagues [29]. Authors created an anti-estrogenic activity score and described a new threshold value of 1798 which was associated with recurrence-free survival (HR: 0 0.67; 95% CI 0.47–0.96). According to the authors, the concordance indices for endoxifen concentrations and this anti-estrogenic activity score were similar. Therefore, the theory of an improved clinical outcome based only on endoxifen concentrations may be appealing, but we certainly think tamoxifen efficacy also relies on other factors than endoxifen concentrations.

A potential limitation of our study might be the number of studied patients. In total, we analysed 662 patients of the CYPTAM study, from whom the endoxifen concentrations and survival information were readily available. In our case, the study population may be underpowered. A post hoc power calculation shows that our study may have approximately 30% of power in order to validate Madlensky’s outcomes. This value is lower than the generally accepted 80% power. However, we also have estimated that nearly 21,500 patients would be required in order to achieve this 80% power with the observed event rate of roughly 8% questioning the clinical relevance of the concentration–effect relationship. In the CYPTAM study design, we assumed an HR of 2.0 in order to calculate the required sample size. However, it might have been an overestimation of the effect size and consequently we cannot exclude an association for a HR ≤ 2.

Another potential limitation of our study might be the implications for the late breast cancer recurrences and the relatively short follow-up duration time (6.8 years (range 0.33–9.34 years)). Late recurrences due to a purely failure of tamoxifen therapy normally happen after 10–15 years of endocrine treatment with tamoxifen [30]. Consequently, the presented results would mainly apply to early-breast cancer recurrences that occurred during tamoxifen therapy. Ideally, this impact of tamoxifen use on (late) breast cancer recurrences should be evaluated in patients who were only treated with tamoxifen and followed for a long time of at least 10–15 years.

Another relevant point in our study might be censoring patients at the time of tamoxifen discontinuation due to a switch to an aromatase inhibitor. Since patients were censored at the moment of switch, it might be difficult to strictly separate the effect and the potential therapeutic failure of tamoxifen from aromatase inhibitors due to a potential carryover effect that would still be present during the therapy with aromatase inhibitors. For instance, if an event takes place after only one or two months of the switch to an aromatase inhibitor, it would more likely to think that this event would be due to a failure to tamoxifen therapy rather than an aromatase inhibitor. However, if an event happens after 18 months of aromatase inhibitor use, this event would be more likely explained by an aromatase inhibitor failure than purely a tamoxifen failure. However, tamoxifen might still have a carryover effect that would be present during the therapy with aromatase inhibitors. To this end, a new endpoint was created and named relapse-free survival complete (RFSc), in which the time of aromatase inhibitor was also included [16]. As previously reported [16], we also did not find any type of differences when comparing the different groups based on the endoxifen threshold concentrations of 5.9 ng/ml (adjusted HR: 1.340; 95% CI 0.788–2.277; *p* value: 0.280). These results suggest that even if there is a carryover effect of tamoxifen, it might still have a minor impact on the clinical survival. Although censoring patients at this point might have its limitations, our outcomes also have the advantage that they are based on the real-world data and represent the consequences of the therapeutic strategy of tamoxifen and aromatase inhibitors.

Another limitation of our study might be due to the fact that endoxifen concentration levels were only collected and measured once during the first year of tamoxifen treatment, either at enrolment and/or after 2 months of tamoxifen therapy in order to assure steady-state concentrations. Although intra-patient variability of endoxifen concentrations is usually considered as low [31], not measuring endoxifen concentrations at some other points in time might be less informative since endoxifen concentrations might change over time. Potential reasons for variations in concentrations might be new concomitant medication, treatment non-adherence and differences between study data and real-world data.

Although the use of TDM in many other drugs in oncology have shown more promising results [32] in order to predict an improved survival outcome based on the drug concentrations, these outcomes obtained from real-world clinical practice may actually question the added value of TDM of tamoxifen efficacy based only on the endoxifen concentrations. In this case, the observed weak exposure relationship between endoxifen concentrations and clinical outcome has a minor effect and consequently, the potential usefulness of TDM might be interpreted of very limited added value for the clinical setting. Therefore, the remaining question in this ongoing controversy might be a proper study design in order to determine the value of TDM based on the endoxifen concentrations in the clinical daily practice. Recently, a few power calculations based on the Madlensky’s study population and the CYPTAM study, suggested that around 1500 patients and 15 years of follow-up would be required in order to adequately investigate this question [33]. In any case, performing such a study might require an important effort. Another approach that could also address this question might be done by combining data of several independent cohorts with available endoxifen concentrations and clinical survival data.

In conclusion, while our analysis shows an endoxifen concentration–effect relationship for relapse and for tamoxifen discontinuation, it does not confirm earlier reported threshold values for the use in TDM nor does it allow definition of a novel threshold. These findings suggest there is a limited added value of TDM to guide tamoxifen dosing.

## Data Availability

The dataset analysed during the current study is available from the corresponding author Prof. Dr. H.J. Guchelaar on reasonable request. These data are not publicly available due to them containing information that could compromise research participant privacy and consent.
